# Thyroid Disorders and Their Impact on Metabolic Syndrome and Cardiovascular Risk: A Narrative Review

**DOI:** 10.7759/cureus.93669

**Published:** 2025-10-01

**Authors:** Arya Raveendran, Sharunan Ragunathan, Joao F Neto, Muram Mustafa Mohamed, Yu Min K Chen, Srijana Baral, Adelyn Mendoza, Waheeda Hatam, Sunil K Yadav, Varthini Karvannan, Shakina Ullagi, Ayoub Kandi, Lalain Masood, Zoya Morani

**Affiliations:** 1 General Practice, Angeles University Foundation, Angeles, PHL; 2 Internal Medicine, Saba University School of Medicine, Saba, BES; 3 Internal Medicine, Universidade do Estado do Rio de Janeiro, Rio de Janeiro, BRA; 4 General Practice, Nile College, Khartoum, SDN; 5 General Practice, International University of Africa, Khartoum, SDN; 6 Internal Medicine, DY Patil University School of Medicine, Navi Mumbai, IND; 7 Medicine and Surgery, Gandaki Medical College, Pokhara, NPL; 8 Internal Medicine, Bayonne Medical Center, Bayonne, USA; 9 Family Medicine, Nangarhar University Medical Faculty, Nangarhar, AFG; 10 Internal Medicine, University of Science and Technology Chittagong, Chittagong, BGD; 11 Internal Medicine, King’s College Hospital, London, GBR; 12 Internal Medicine, Baba Farid University of Health Sciences, Faridkot, IND; 13 Medicine, University of Algiers, Algiers, DZA; 14 Dermatology, Bahria University Medical and Dental College, Karachi, PAK; 15 Family Medicine, Washington University of Health and Science, San Pedro, BLZ

**Keywords:** hyperthyroidism and cardiovascular risk, hyperthyroidism and metabolic syndrome, hypothyroidism and cardiovascular risk, hypothyroidism and metabolic syndrome, thyroid disorders and cardiovascular risk, thyroid disorders and metabolic syndrome

## Abstract

Thyroid function regulates numerous processes that influence metabolism and maintenance of homeostasis. Its dysfunction can adversely affect multiple clinical parameters. Notably, thyroid disorders have been correlated with metabolic changes and increased risk of cardiovascular disease. In both cases of hyperthyroidism and hypothyroidism, thyroid hormone interrupts insulin sensitivity and adipocyte metabolism, leading to abnormal glucose metabolism, lipid profile, and cardiometabolic health. As thyroid disorders represent modifiable risk factors, including reduced physical activity, cigarette smoke, mean body mass index, and alcoholism, a thorough understanding and up-to-date knowledge of how these conditions affect different systems may provide clinicians with a better opportunity for early detection, which contributes to reducing the progression of health complications and improving both quality of life and life expectancy. This narrative review aims to identify and summarize current understanding of the relationship between thyroid disorders and their effect on metabolic syndrome and cardiovascular risk, including the possible pathophysiology, and discuss the associated correlations. To achieve this, a detailed search was conducted using credible databases such as PubMed and Google Scholar. We also used sidebar filters to include articles relevant to our topic and critically analyzed them before citing the references in this review.

## Introduction and background

**Figure 1:**
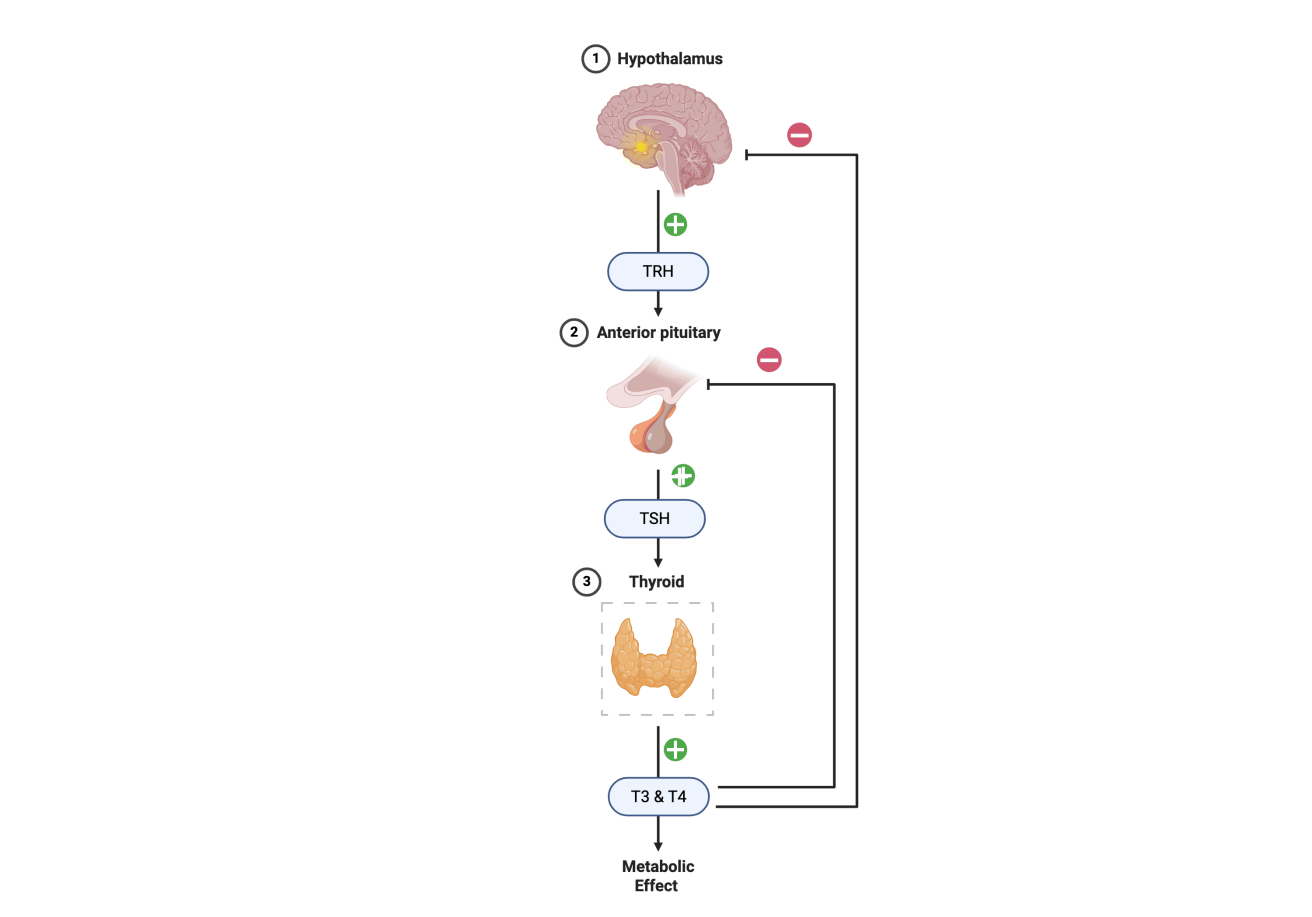
The hypothalamic-pituitary-thyroid axis. This figure was created by the author Sharunan Ragunathan using Biorender, accessed on 05/08/2025 [4]. HPT = hypothalamic pituitary thyroid; TRH = thyroid-releasing hormone; TSH = thyroid-stimulating hormone; T3 = triiodothyronine; T4 = thyroxine

Nearly 200 million people worldwide suffer from thyroid disorders, with a growing number due to autoimmunity. An increasing cohort of studies describes the role of the thyroid in maintaining physiological homeostasis and the link between thyroid disorders and clinical conditions such as cardiovascular disease (CVD), diabetes mellitus (DM), cancer, depression, and others. Understanding the physiology of thyroid hormones (THs) is essential to perceive how these correlations have been established [1,2]. The thyroid gland produces two principal hormones, thyroxine (T4) and triiodothyronine (T3), which are regulated by the hypothalamic-pituitary-thyroid (HPT) axis. Thyrotropin-releasing hormone (TRH), which is produced by the hypothalamus, stimulates the anterior pituitary gland to release thyroid-stimulating hormone (TSH). In response to TSH stimulation, the thyroid gland generates T4 and T3 through the hypophyseal portal system. The HPT axis is maintained using a negative feedback loop. Changes in T3 and T4 levels provide input to the hypothalamus and pituitary glands, which regulate TRH and TSH synthesis [3]. Although T4 is the most abundant form produced by the thyroid gland, T3 is the physiologically active hormone, mostly synthesized peripherally by deiodinases [4]. Figure 1 outlines the HPT axis.

The thyroid function test (TFT), which measures TSH and free T4 (FT4) levels, reflects the thyroid gland’s status. A decrease in TSH is seen in hyperthyroidism, while it is increased in hypothyroidism. The opposite is true for FT4 levels, where high FT4 levels are seen in overt hyperthyroidism, and low levels are seen in overt hypothyroidism. Within the normal range, FT4 is found in subclinical hypo- and hyperthyroidism [5]. Another thyroid disorder known as low triiodothyronine syndrome (LT3S), also referred to as nonthyroidal illness syndrome or euthyroid sick syndrome, is characterized by a decline in both total and free T3 (FT3) levels while levels of T4 and TSH in the blood remain within the reference range, and reverse T3 (rT3) levels increase [6].

Thyroid dysfunction affects several metabolic clinical parameters, such as blood pressure (BP), glucose and lipid metabolism, and cardio-endothelial function, suggesting a link between thyroid disorders and metabolic syndrome (MetS) [7]. MetS is characterized by the co-occurrence of three or more of the following: abdominal obesity, atherogenic dyslipidemia, elevated BP, and insulin resistance [8]. As such, patients with hypothyroidism suffer from abdominal obesity, leading to insulin resistance [9]. Moreover, hypothyroidism increases mortality rates in people with CVD, due to impairment of myocardial contractility. Likewise, those with LT3S and hyperthyroidism are at greater risk for atrial fibrillation (Afib) and angina due to coronary artery vasospasm [6,10,11]. These findings suggest the possible correlation between thyroid diseases and both metabolic and cardiovascular function.

This review aims to analyze the impact of thyroid dysfunction on MetS and cardiovascular risk. Multiple studies, including review articles, clinical trials, case reports, and observational studies, were analyzed to better comprehend these effects. Only English-language articles published in the last decade were included; however, research focusing on thyroid issues during pregnancy or on children was not considered.

## Review

Thyroid hormones and metabolic homeostasis

THs significantly impact metabolic homeostasis at the nuclear level via two mechanisms: genomic and non-genomic pathways [12]. The genomic pathway implies that THs act on the nucleus first, binding to specific receptors and leading to gene expression. In contrast, in the non-genomic pathway, THs act peripherally on blood vessels and cardiac monocytes, where transcription factors do not regulate transcription [6].

The attachment of the TH to nuclear receptors leads to the expression of proteins that regulate basal metabolic rate (BMR), which is one of its primary effects [13]. Hence, BMR can be increased by inducing the activity of Na+/K+ adenosine triphosphatase (ATPase), enhancing mitochondrial synthesis, and promoting mitochondrial uncoupling in the liver, muscle, and brown adipose tissue. Due to this elevated activity, heat production increases, leading to increased energy expenditure and thermogenesis within the body. This suggests that hyperthyroid patients experience heat intolerance, while hypothyroid patients experience cold intolerance [14].

Studies show that THs modulate carbohydrate metabolism via gluconeogenesis and glycogenolysis in the liver. Gluconeogenesis is augmented by rate-limiting enzymes glucose-6-phosphatase and phosphoenolpyruvate carboxykinase. In contrast, glycogenolysis is influenced by lysosomal alpha-glucosidase and hepatic phosphorylase kinase [15]. With regard to lipid metabolism, THs stimulate lipogenesis, oxidation of fatty acids, and increase the rate of cholesterol clearance, making individuals with hypothyroidism prone to hypercholesterolemia [15]. However, it should be noted that THs’ control over metabolism depends on several other factors, such as age, circadian rhythm, genetics, and iodine uptake [12].

Thyroid dysfunction as a modulator of metabolic syndrome

Thyroid dysfunction and MetS share a mutual relationship and often go hand-in-hand. About 20% of the adult population presents with thyroid dysfunction and MetS in clinical practice. [16]. The four major components of MetS that are affected by thyroid dysfunction include (1) body adiposity, that is, central and measured by waist circumference; (2) serum glucose levels, that reflect DM; (3) lipid profile, that is, serum triglycerides (TGs) or high-density lipoprotein cholesterol (HDL-C); and (4) BP levels [16].

**Figure 2:**
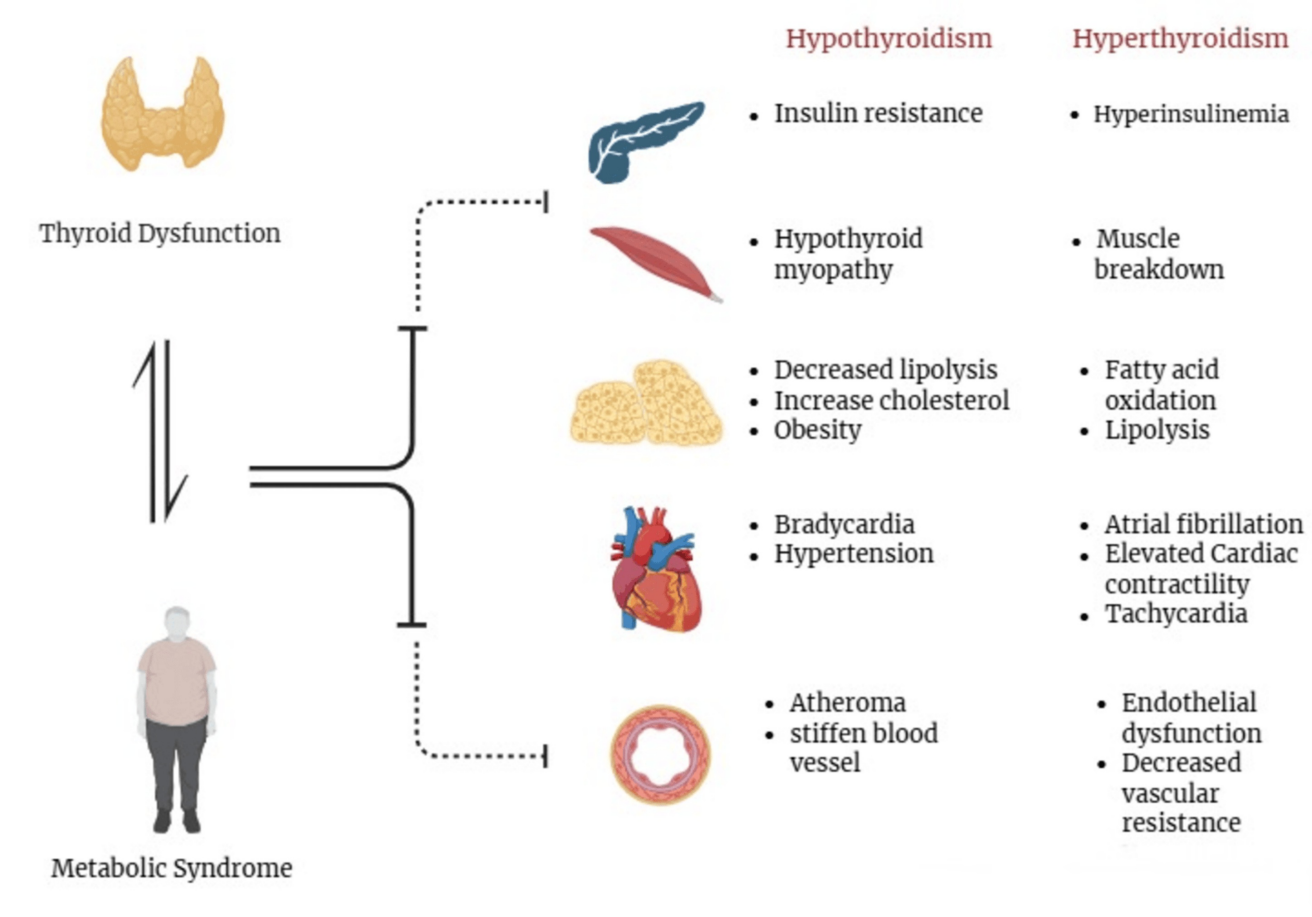
The effect of thyroid dysfunction and metabolic syndrome on different organs. This figure was created by the author Arya Raveendran using Biorender, accessed on 05/12/2025 [17,18].

THs and their metabolites are powerful regulators of BMR. However, the relationship between TH and adiposity is not unidirectional; that is, increased adipocytes within the body may secrete several hormones or cytokines that may influence thyroid function [16]. Additionally, TSH acts directly on adipose tissue, inducing lipolysis and inhibiting insulin secretion via protein kinase B phosphorylation, resulting in insulin resistance. Thyroid dysfunction is also associated with glucose metabolism, as TRβ receptors are present in the liver, and glucose transporter 4 in skeletal and adipose tissue. T3 regulates these receptors for hepatic gluconeogenesis, glycogen metabolism, and insulin signalling [16]. Lipid abnormalities associated with MetS are hypertriglyceridemia and low serum HDL-C, which can be attributed to alterations in thyroid function. THs stimulate both lipid production and degradation via lipogenesis and lipolysis; it also stimulates fatty acid oxidation. Sometimes, failure in cholesterol clearance may lead to atherosclerosis of the vessels [16]. Lastly, blood vasculature and heart are mediated by THs, via thyroid receptor-mediated gene regulation or another nonclassical pathway at the cytoplasmic and cellular membrane level. TH nuclear receptors are present in myocytes and blood vessels; these receptors include TRα and TRβ, which lead to positive or negative regulation in cardiac function and vascular resistance. Overall, TSH hinders multiple components of MetS, including insulin resistance, lipid control, adipose tissue, and hypertension. These interferences usually occur at the cellular level and manifest clinically, leading to higher-risk cardiovascular events, as seen in Figure 2 [17].

Subclinical thyroid disorders: a silent contributor to cardiometabolic risk

Subclinical thyroid disorders, defined by abnormal TSH levels and normal FT4, are silent yet significant disorders that contribute to cardiometabolic risk by causing dyslipidemia, vascular dysfunction, and cardiac complications. Subclinical hypothyroidism increases cardiometabolic risk through lipid alterations and vascular impairment. Elevated TSH levels raise low-density lipoprotein-cholesterol (LDL-C) and TGs, promoting atherosclerosis [19]. This is backed up by a meta-analysis that found an increase in LDL levels of 10-20% compared to euthyroid controls [20]. These changes increase arterial stiffness, heightening the risks of hypertension and coronary artery disease (CAD) [21]. Mechanisms also include reduced lipoprotein lipase activity and systemic inflammation, which exacerbate cardiometabolic burden.

Subclinical hyperthyroidism primarily disrupts cardiac rhythm and function; that is, low levels of TSH are associated with a 2-3-fold increase in Afib risk, a significant risk factor for stroke [22]. This arrhythmia stems from excess TH, accelerating heart rate and promoting left ventricular hypertrophy (LVH) [23]. Overall, subclinical hyperthyroidism elevates cardiovascular risks, including heart failure (HF) and stroke, particularly in older adults [21]. These effects highlight the need for early detection to prevent silent cardiac complications. Both disorders amplify cardiometabolic risk across populations; however, these disorders cause a 20-80% cardiovascular mortality risk to those with comorbidities such as DM or hypertension [21]. The asymptomatic nature of these conditions often delays diagnosis, as routine TSH screening is not universally available [19]. Treatment remains debated as managing these disorders is complex due to their variable risk profiles [23]. Due to their silent contribution to cardiometabolic disease, subclinical thyroid disorders warrant increased attention. Targeted TSH screening in high-risk groups, such as those with dyslipidemia or arrhythmias, could facilitate early intervention [22]. Clinicians and public health strategies should prioritize awareness and research into optimal treatment thresholds to reduce cardiovascular and metabolic risks.

Table 1 summarizes recent research findings on the cardiometabolic risks linked to subclinical and other thyroid disorders. By highlighting the key studies, with their associated cardiovascular outcomes (e.g., atherosclerosis, Afib, and mortality), and their mechanistic implications (e.g., lipid dysregulation, endothelial dysfunction), it provides a comprehensive overview to inform clinical understanding, guide risk assessment, and underscore the need for targeted screening and management strategies in affected populations.

**Table 1:** The cardiometabolic risk and mechanism associated with different thyroid disorders. This table was created by the author Adelyn Mendoza using Google Microsoft on 5/8/25 [20-24]. LDL = low-density lipoprotein; LDL- C = low-density lipoprotein cholesterol; TGs = triglycerides; LT4 = levothyroxine; Afib = atrial fibrillation; TH = thyroid hormone; HR = heart rate; LVH = left ventricular hypertrophy; CV = cardiovascular

Disorder	Cardiometabolic risk	Mechanism or implication
Hypothyroidism	Atherosclerosis, hypertension	Raised LDL-C and TGs promote vascular damage
Hypothyroidism	Increased LDL (10–20%)	Reduced lipoprotein lipase activity; LT4 may normalize lipids
Hypothyroidism	Coronary artery disease	Endothelial dysfunction and arterial stiffness increase cardiovascular risk
Hyperthyroidism	Afib (2–3-fold risk)	TH excess accelerates HR, raising stroke risk
Hyperthyroidism	Heart Failure, stroke	LVH drives cardiac complications
Both	20–80% higher CV mortality	Inflammation and cardiac overload necessitate screening in high-risk groups

Cardiovascular consequences of thyroid dysfunction

THs are essential for regulating metabolism and maintaining the structural integrity and functional performance of various organs, including the cardiovascular system. Both hypothyroidism and hyperthyroidism can lead to alterations in cardiac and endothelial function, contributing to atherosclerotic plaque formation, dyslipidemia, and elevated BP. Even a slight change in TH levels can modulate cardiovascular function by acting on the specific receptors within the heart and blood vessels [6].

**Figure 3:**
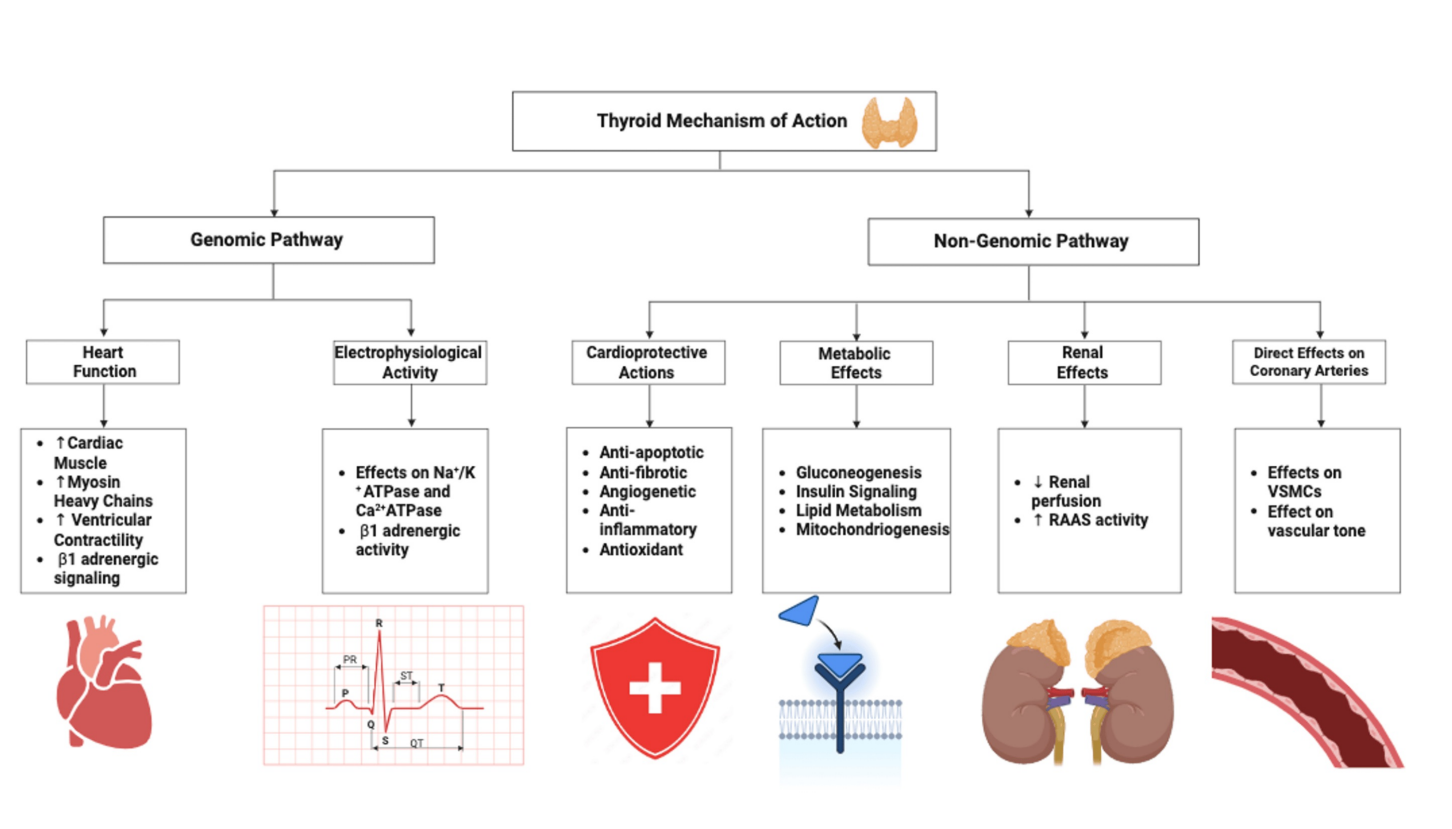
The effects of thyroid hormones on the cardiovascular system This figure was made by the author Sharunan Ragunathan using Biorender, accessed on 05/10/2025 [6]. RAAS = renin-angiotensin-aldosterone system; VSMCs = vascular smooth muscle cells; ATPase = adenosine triphosphatase

Under resting conditions, TH typically enhances cardiac output by increasing cardiac contractility and heart rate, improving both systolic and diastolic function, and lowering systemic vascular resistance. Consequently, even a subtle hormonal imbalance may raise the risk of CVDs as these hormones act via genomic and non-genomic pathways, modulating the autonomic nervous system and renin-angiotensin-aldosterone system, which are key regulators of the circulatory dynamics, as seen in Figure 3 [6].

Studies stipulate that plasma levels of TH cause a shift in the hemostatic system; that is, low plasma levels of TH create a hypocoagulable and hyperfibrinolytic state. In contrast, increased levels of TH create a hypercoagulable and hypofibrinolytic state, indicating an increased risk of bleeding in hypothyroidism and an elevated risk of thromboembolism in hyperthyroid patients. An acquired von Willebrand disease is more common in hypothyroid patients, whereas levels of FT4, fibrinogen, factor VIII, and von Willebrand factor gradually rise, potentially causing symptomatic thromboembolism in hyperthyroid patients [25].

Hyperthyroidism is associated with thyrotoxic cardiomyopathy, a condition marked by Afib, left ventricular hypertrophy, and impaired diastolic function that may ultimately progress to HF. Notably, HF occurs in approximately 8% of patients with hyperthyroidism, with Afib being the leading contributing factor. In some cases, if left untreated, hyperthyroidism can result in pulmonary hypertension and angina-like symptoms [26,27].

On the other hand, hypothyroidism may impede cardiac relaxation, leading to a reduction in myocardial contractility, especially during diastole. This is known to be associated with diastolic hypertension and, in specific individuals, may even coexist with CAD, further damaging myocardial function. As T3 is crucial for gene regulation in cardiac muscle, reduced T3 levels can adversely affect myocardial remodeling and contraction. In patients with existing heart disease, decreased levels of free T3 have been correlated with increased mortality rates [6]. Subclinical hypothyroidism, despite its benign clinical course, has been associated with hypertension, dyslipidemia, and endothelial dysfunction [28,29]. Moreover, it has been proven to be a predictive factor of all-cause mortality and cardiovascular events [30]. On the other hand, subclinical hyperthyroidism has been a contributing factor to arrhythmia and ventricular diastolic dysfunction [29]. Studies have further revealed that both subclinical hypo- and hyperthyroidism increase the risk of venous thromboembolism; therefore, routine screening is recommended in high-risk patients [31].

Inflammation and adipokine imbalance: shared pathogenic axis

Adipokines, produced by adipocytes, have various effects on our body, such as modulating cardiovascular, endocrine, metabolic, reproductive, immune, and inflammatory functions, along with several other physiological functions. Adipokines can be broadly categorized into pro-inflammatory and anti-inflammatory, as shown in Table 2.

**Table 2:** The different types of proinflammatory and anti-inflammatory adipokines. This table was created by the author Arya Raveendran using Microsoft Word on 05/08/2025 [32]. CTRP = C1q/tumor necrosis factor-related protein; RBP 4 = retinol binding protein 4; SFRP 5 = secreted frizzled-related protein 5

Proinflammatory adipokines	Anti-inflammatory adipokines
Leptin	Adiponectin
Chemerin	CTRP
Resistin	Omentin
RBP 4	SFRP 5

**Figure 4:**
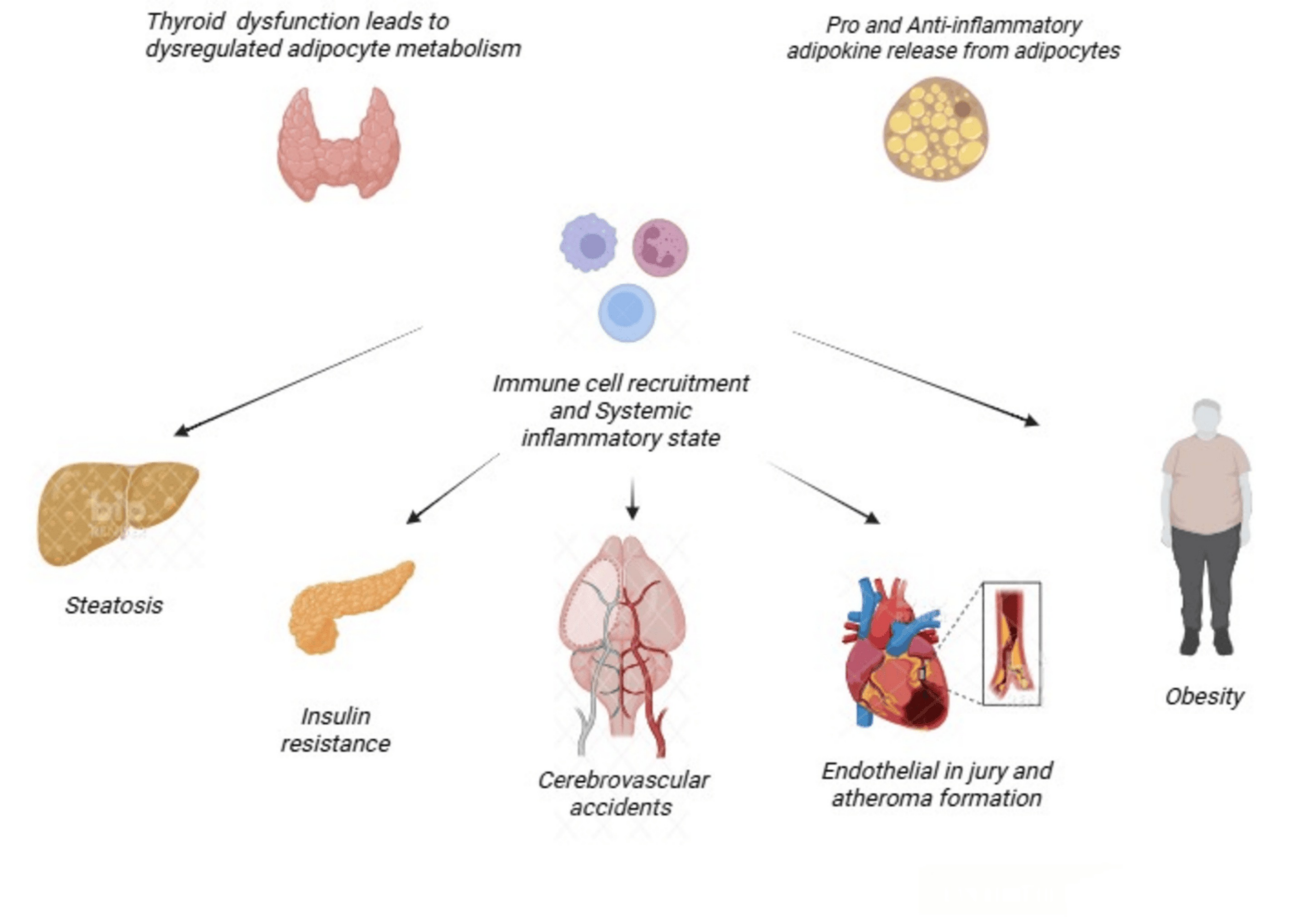
The effect of adipokines on different organ systems. This figure was created by the author Arya Raveendran using Biorender, accessed on 05/09/2025 [32].

Adipose tissue is composed of several cells, such as adipocytes, pre-adipocytes, fibroblasts, mesenchymal cells, B-cells, T-cells, macrophages, natural killer (NK) cells, dendritic cells, neutrophils, and eosinophils. The activation and proliferation of these cells depend on the circulating cytokines and adipokines in fat tissues. Hence, depending on the pro- and anti-inflammatory pathways, adipokines are involved in the pathogenesis of various metabolic diseases [32]. Studies have also shown that pro-inflammatory cytokines and adipokines are linked to a greater risk of CVD, as the cytokines released from the immune cells result in vascular endothelial damage, enhanced arterial rigidity, inflammation, lipolysis, oxidative stress, thrombosis, and atherosclerotic plaque formation, leading to CVD, as shown in Figure 4.

Adipokines such as leptin, a satiety hormone, are found to be elevated in systemic inflammatory states. Leptin enhances the levels of interleukin-6, 8, 12, 18, and tumor necrosis factor-α in the circulation, contributing to the inflammatory state. It is also a key component associated with platelet activation, enhanced tissue factor and adhesion molecule expression, leading to narrowing of the vascular lumen [24]. Resistin and chemerin, other proinflammatory adipokines, play an important role in angiogenesis and inflammation, whereas adiponectin, an anti-inflammatory adipokine, reduces cardiovascular risk. This increases vascular endothelial nitric oxide release, leading to vasodilation, decreased endothelial damage, oxidative stress, and atherosclerosis formation [32]. Hence, reduced adiponectin is associated with increased risk of cardiovascular diseases. This showcases that TH alters metabolic pathways of adipocytes, leading to MetS.

Reconsidering treatment thresholds in borderline thyroid disease

In subclinical hypothyroidism, the upper limit of TSH is the 97.5th percentile (4-5 mU/L). Studies have shown that lowering the upper limit to 2.5 mU/L results in overtreatment of patients, which causes unnecessary harm. Current best practice recommends treatment based on TSH levels, symptoms, patient status, and the presence of thyroid autoantibodies [33].

Subclinical hypothyroidism carries the potential risk of progression into overt hypothyroidism. Hence, periodic assessment of patients with hypothyroidism is necessary to prevent complications such as MetS, CVD, stroke, dyslipidemia, bone health, and pregnancy-related complications. Research shows that nearly half of the cases with subclinical hypothyroidism resolve spontaneously in patients with a TSH level in the range of 4-6 mU/L [34].

**Figure 5:**
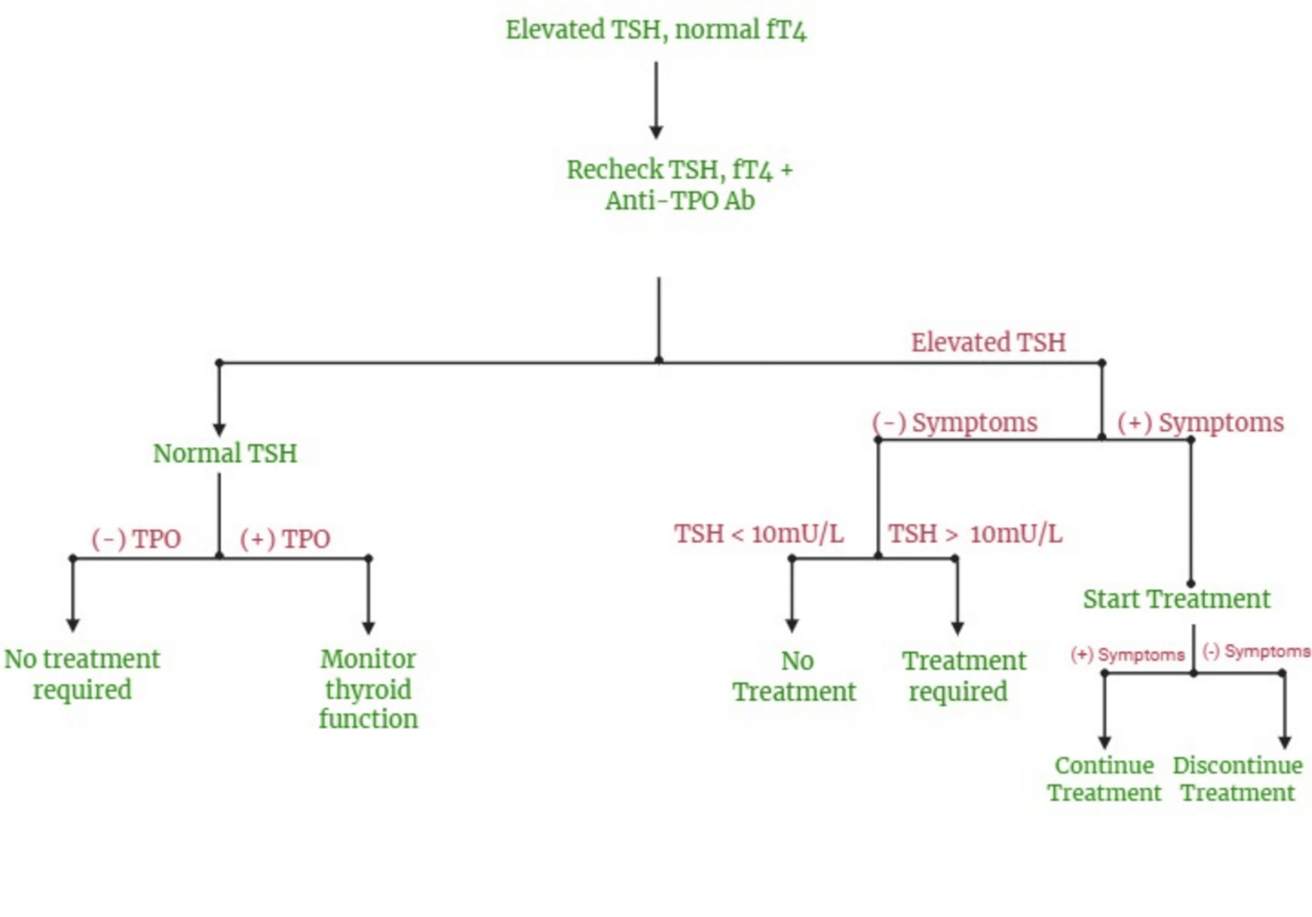
The management of subclinical hypothyroidism. This figure was created by the author Arya Raveendran using Biorender, accessed on 09/05/2025 [35]. TSH = thyroid-stimulating hormone; fT4 = free thyroxine; Anti-TPO Ab = anti-thyroid peroxidase antibody; TPO = thyroid peroxidase

If TSH exceeds the normal range, FT4 should be assessed to exclude overt hypothyroidism, which requires treatment with levothyroxine (LT4). In patients with subclinical hypothyroidism, TSH levels should be monitored annually, and treatment is required if TSH levels are >10 mU/L. Patients are treated with LT4 if TSH levels are <10 mU/L with any of the following present: symptoms favouring clinical hypothyroidism, goitre, strong family history of autoimmune disease, positive anti-TPO autoantibodies, presence of atherosclerotic CVD, HF, and pregnancy [34]. Recent studies have shown that subclinical hypothyroidism is linked to CVD, HF, and death. However, patients with TSH levels between 5-10 mU/L remain controversial [35]. Figure 5 outlines the management for subclinical hypothyroidism.

Subclinical hyperthyroidism can be classified as type I with a TSH level between 0.1 and 0.4 mU/L and type II with a TSH level <0.1 mU/L [36]. According to the American Thyroid Association, patients <65 years of age require monitoring of thyroid function every 6-12 months. Treatment is indicated if TSH <0.1 mU/L, symptomatic, persistent elevation of thyrotropin receptor antibody titer, Afib, and osteoporosis. Patients aged >65 years, with comorbidities, require treatment for subclinical hyperthyroidism, due to the increased risk of CVDs and osteoporosis [35]. Before initiating treatments for subclinical hyperthyroidism, TSH levels should be rechecked after three to six months to confirm the persistence of subclinical hyperthyroidism. Subclinical hyperthyroidism may result from self-limiting conditions such as virus-induced thyroiditis, which may result in transient thyroiditis or gradual resolution within a few months. Persistent low TSH levels could be due to hypothalamic or pituitary stimulation, exogenous or iatrogenic, thyroiditis, Graves’ disease, nodular goiter, and cancer. Treatment modalities for hyperthyroidism depend on the underlying etiology [37].

Indications for the treatment of subclinical hyperthyroidism include a TSH value <0.1 mIU/L, history of Afib, fractures, and overt thyrotoxicosis. Patients with subclinical hyperthyroidism secondary to exogenous LT4 intake can be managed by lowering the prescribed LT4 dosage. Whereas, treatment of endogenous subclinical hyperthyroidism can be treated by antithyroid medications such as methimazole, propylthiouracil, radioactive iodine uptake therapy (RIU therapy) for multinodular goitre, and surgical resection or thyroidectomy for malignancy [37]. After the initiation of antithyroid medication, TSH should be repeated within two to six weeks to adequately adjust the dosage of medication. Once euthyroid, the antithyroid medication dose may be reduced to 30-50% of the initial strength, with further adjustments every four to six weeks based on TSH levels. Patients on long-term treatment with antithyroid medications should monitor their TSH levels every two to three months [38].

Clinical management toward an integrated endocrine cardiometabolic approach

The triad of endocrine, cardiovascular risk, and MetS raises significant obstacles in medicine, as these conditions share a similar way of functioning [38]. Initially used for glycemic control, sodium-glucose cotransporter-2 inhibitors, glucagon-like peptide-1 receptor agonists (GLP-1RAs), and dual gastric inhibitory peptide/GLP-1RAs are demonstrating crucial benefits in cardiometabolic diseases [38].

A few recent studies have shown that integrated management, including lifestyle medicine, significantly focuses on behavioral risk factors to improve long-term habits and prognosis [39]. In nutrition, diets such as the Mediterranean or DASH (Dietary Approaches to Stop Hypertension) have effectively lowered BP and LDL-C by 10%, improving glycemic control and reducing cardiovascular risk [39]. Reducing sodium intake to less than 2 g per day and increasing potassium intake by 0.5-1.0 g per day has significantly reduced systolic BP. However, caution is necessary in patients with chronic kidney disease on potassium-sparing medications [38]. Furthermore, physical activity such as aerobic exercise has been shown to reduce BP (by 7-8 mmHg systolic and 4-5 mmHg diastolic). A 5% weight loss in weight management, a disciplined sleep routine, and smoking and alcohol cessation are said to have significant health benefits. These patient-centered approaches have allowed individuals to have longer life spans [39].

Management of thyroid dysfunction begins with TFTs; patients with CVD are initially started on low-dose LT4, diuretic, and β-blockers with follow-up every 10-12 weeks [6]. β-blockers are recommended for patients with Afib and supraventricular tachycardia to control the heart rate. With the administration of antithyroid medication, adding potassium iodide via the oral route or sodium iodide via the intravenous route can help suppress the release of pre-existing TH reserves [6]. For individuals who cannot tolerate antithyroid medication, thyroidectomy is the ideal treatment of choice [6]. LT4, known for its economical and effective drug status for hypothyroidism, is used to achieve a euthyroid state, without increasing the peripheral vascular resistance, but improving myocardial perfusion. Overall, individuals are recommended a personalized structured management according to their individual case, but Cardiovascular-Endocrine-Metabolic Medicine is rather enhancing and still a developing field [39].

Screening and early detection: an evolving paradigm

Thyroid disorders, particularly subclinical hypothyroidism, often have a silent presentation. With stronger evidence linking thyroid dysfunction, MetS, cardiovascular risk, early screening, and detection have become critical in preventive care. The cost-effectiveness of screening for mild thyroid dysfunction has been well appreciated, along with early treatment that could yield improvements in lipid profile, cardiac structure, function, and thereby impact metabolism and cardiovascular risk [20]. Recent advances in genetic studies, such as the emergence of genome-wide association studies, have proven instrumental in identifying genetic variants associated with TH regulation, including TSH, FT4, FT3, and TT3 levels. It revealed specific single-nucleotide polymorphisms tied to thyroid function, paving the way for personalized risk assessments and targeted prevention strategies for thyroid-related metabolic disorders [5]. In parallel, retrospective clinical studies have highlighted the relationship between TSH suppression and cardiovascular outcomes, particularly in patients treated with LT4 or those who have undergone radioactive iodine therapy. These findings suggest that the extent of TSH suppression could be a meaningful indicator of cardiovascular risk, emphasizing the need for individualized TSH goals in long-term thyroid management [40]. Clinical parameters, such as BMI, also contribute to early detection as elevated BMI warrants further metabolic and hormonal evaluations, which include TFTs, insulin resistance markers (e.g., fasting glucose, homeostatic model assessment of insulin resistance), and lipid profiles. In some cases, biomarkers such as leptin and adiponectin are being explored to understand the relationship between adipose tissue function and thyroid activity [40].

Moreover, a comprehensive assessment is crucial in individuals presenting with unexplained cardiovascular symptoms. This involves evaluating TH levels alongside catecholamines, especially in suspected cases of pheochromocytoma, and screening for thyroid autoantibodies. When indicated, imaging techniques such as echocardiography or cardiac MRI can aid in identifying thyroid-induced myocardial dysfunction [41]. Since thyroid dysfunction often overlaps with MetS, routine TSH screening is becoming important for early detection in people with MetS. Early identification of subclinical hypothyroidism in these patients can also help prevent it from progressing to overt hypothyroidism and reduce long-term cardiovascular risks. As a result, TFT is now considered a standard component of metabolic evaluations [2].

Demographic and population-specific variability 

**Figure 6:**
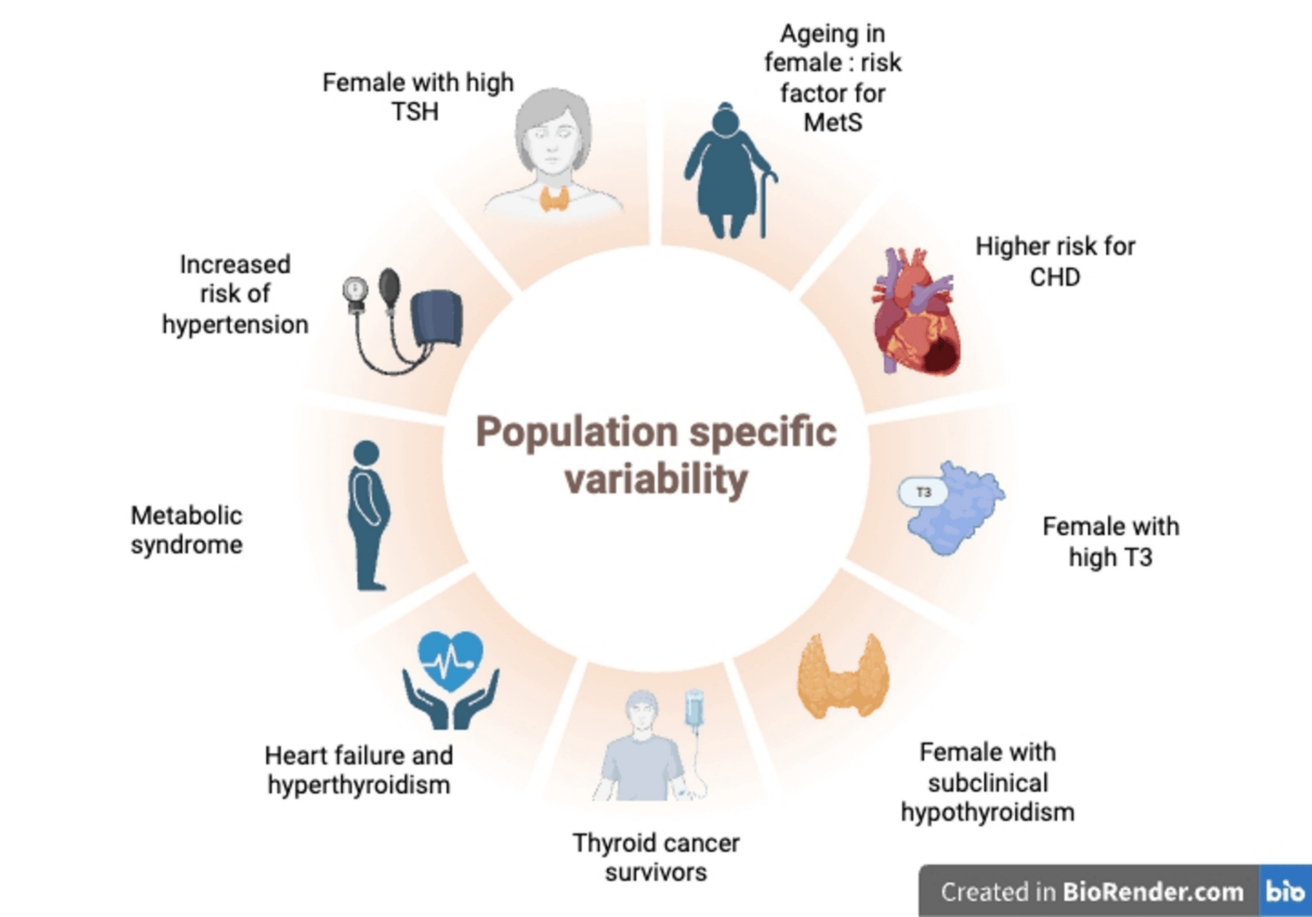
The population variability regarding thyroid dysfunction, MetS, and CVD. This figure was created by the author Srijana Baral using Biorender, accessed on 09/05/2025 [26,28,42-44]. TSH = thyroid-stimulating hormone; T3 = triiodothyronine; CHD = coronary heart disease; MetS = metabolic syndrome

Gender and age substantially influence thyroid function and MetS; that is, studies have shown that females are at a higher risk for MetS due to relatively higher TSH and FT3 levels. [42]. Additionally, middle-aged females with subclinical hypothyroidism are at greater risk for hypertension when compared to older females [28]. Hyperthyroidism, otherwise, has been reported to increase the occurrence of HF, compared to the general population; having thyroidectomy as the most successful treatment to reduce HF prevalence [26]. Concerning neoplastic processes, patients who were younger when diagnosed with thyroid cancer (<65 years old) may have a reduced risk of chronic heart disease when compared to patients diagnosed at later ages [43]. Overall, thyroid dysfunction does not affect everyone similarly. Recognizing population-specific differences is crucial for accurate diagnosis and improved treatment strategies. Figure 6 describes population-specific variability with regard to thyroid dysfunction, MetS, and CVD.

Research gaps and future directions

Multiple studies have suggested a strong association between elevated TSH levels and metabolic syndrome, but a causal relationship has not been established [45]. This relationship needs to be studied through longitudinal studies with a larger population, which helps researchers control factors that change over time and attribute changes in health (metabolic syndrome) to specific causes (elevated TSH). Moreover, a randomized controlled trial (RCT) is necessary to establish causality by controlling the variable of interest (TSH level) and determining the direct effect on metabolic syndrome.

While the normal TSH reference range is commonly accepted as 0.4 to 4.0 mIU/L in healthy adults, clinical experience and research suggest the existence of an “optimal range,” which is narrower than the TSH reference range. A large meta-analysis supports this finding, as it has shown decreased risk for CVD and mortality was observed in individuals with TSH levels between 1.9-2.9 mIU/L (60th-80th percentiles) [46]. This implies that TSH values outside this interval have an increased risk for adverse cardiovascular outcomes. Future studies should focus on determining a more precise reference range, which will facilitate treatment decisions and the identification of patients who will benefit from early therapeutic intervention.

Expanding on that, an important research question that needs to be answered is whether early therapeutic intervention in subclinical thyroid disease can reduce long-term cardiovascular morbidity and mortality. A high-quality RCT called the Trial of Radical Upfront Surgical Therapy (TRUST) has shown no significant benefit from levothyroxine therapy in patients over 65 years with subclinical hypothyroidism [47]. However, it remains uncertain for young patients (<65 years old). Therefore, to better answer this question, it is preferable to rely on observational cohort studies, as they are easier to implement by following a large cohort of untreated vs. treated young adults with subclinical hypothyroidism over time to assess differences in cardiovascular outcomes. Additionally, performing a meta-analysis targeting young patients will enhance statistical power and provide strong assertions about the benefits of early intervention in this population. Ultimately, RCTs are the gold standard for proving a definitive answer.

Limitations

The main limitation of this review article is the selected study population, which does not include childbearing mothers and children with thyroid dysfunction. The review also does not follow the strict PRISMA guidelines used for systematic reviews. Moreover, it included recent English-language studies done between 2015 and 2025. This review does not address genetic determinants or medication benefits in metabolic and CVD, which could impact outcomes. Further, as this is a narrative review article, no new statistical analysis was performed for this review; rather, formerly published meta-analyses were synthesized to reinforce the analysis presented herein.

## Conclusions

This review highlights the critical impact of thyroid disorders on the development of MetS and CVD. Thyroid dysfunction, by its influence on lipid profile, insulin sensitivity, and vascular dysfunction, contributes to increased cardiovascular risk and MetS. Understanding these associations ensures timely diagnosis and management and recognizes thyroid dysfunction as a modifiable risk factor, enabling clinicians to identify the root cause of the disease (early screening). This leads to better prevention and mitigation strategies, thereby reducing the progression of health complications and prolonging lifespan.
